# Canine olfactory detection and its relevance to medical detection

**DOI:** 10.1186/s12879-021-06523-8

**Published:** 2021-08-19

**Authors:** Paula Jendrny, Friederike Twele, Sebastian Meller, Albertus Dominicus Marcellinus Erasmus Osterhaus, Esther Schalke, Holger Andreas Volk

**Affiliations:** 1 Department of Small Animal Medicine and Surgery, University of Veterinary Medicine Hannover, Bünteweg 9, 30559 Hannover, Germany; 2 Research Center for Emerging Infections and Zoonoses, University of Veterinary Medicine Hannover, Bünteweg 17, 30559 Hannover, Germany; 3 Bundeswehr School of Dog Handling, Gräfin-Maltzan-Kaserne, Hochstraße, 56766 Ulmen, Germany

**Keywords:** Biomedical detection dogs, Olfaction, Olfactory sense, Screening method, Sniffer dogs

## Abstract

The extraordinary olfactory sense of canines combined with the possibility to learn by operant conditioning enables dogs for their use in medical detection in a wide range of applications. Research on the ability of medical detection dogs for the identification of individuals with infectious or non-infectious diseases has been promising, but compared to the well-established and–accepted use of sniffer dogs by the police, army and customs for substances such as money, explosives or drugs, the deployment of medical detection dogs is still in its infancy. There are several factors to be considered for standardisation prior to deployment of canine scent detection dogs. Individual odours in disease consist of different volatile organic molecules that differ in magnitude, volatility and concentration. Olfaction can be influenced by various parameters like genetics, environmental conditions, age, hydration, nutrition, microbiome, conditioning, training, management factors, diseases and pharmaceuticals. This review discusses current knowledge on the function and importance of canines’ olfaction and evaluates its limitations and the potential role of the dog as a biomedical detector for infectious and non-infectious diseases.

## Background

Canines are macrosmatics with an extraordinary olfactory sense and memory [1, 2]. Olfaction is mandatory for the dog to perceive environmental information, which has been used successfully by humans for tracking and detection of pest and prey animals and other food sources [3]. Routinely, dogs nowadays are predominantly deployed for the identification of explosives, drugs, currencies, people, endangered animal species and parasites [4]. In recent years, medical scenting dogs have been trained to detect different medical conditions, but this area of work is still relatively in its infancy [5]. The use of odour detection as a diagnostic tool is of increasing interest in recent [5, 6]. This review will summarise information on odour origin and composition, neuroanatomy and physiology of the canine olfaction, different impacts on the olfactory sense and majorly current research outcomes critically evaluating the possible role of the dog as a biomedical detector.

## Methods

Search strategies for this review included electronic search engines for publication databases, searching reference lists of published papers and information from relevant scientific conferences and discussion groups. For the section about biomedical detection dogs three databases (Google Scholar, Science Direct and PubMed) were searched for studies reporting the training, testing and deployment of biomedical detection dogs between 2004 and 2021. The searches were performed by the authors using the following keywords: “biomedical detection dogs” or “detection dogs” or “canines” in combination with “infectious diseases”, “non-infectious diseases”, “malaria”, “SARS-CoV-2”, “COVID-19”, “hypoglycaemia”, “epileptic seizure”, “cancer”, “bacteria” or “viral”. Peer-reviewed studies and pre-prints published in English and results presented at the WHO R&D Blueprint COVID-19 consultation [6] were evaluated. Literature that addressed detection rate and/or diagnostic accuracy (sensitivity and specificity) of biomedical detection dogs without restrictions to year of publication was included in this review article. Only double-blinded and randomised studies for SARS-CoV-2 detection were reported in this study.

## Main text

### Research on biomedical detection dogs

The use of biomedical detection dogs for various infectious and non-infectious diseases like Helicobacter pylori [7], different cancer types [8–17], hypoglycaemia in diabetes mellitus patients [18–20], epileptic seizures [21], bacteriuria [22], bovine virus diarrhoea [23], COVID-19 [24–33], Malaria [34] and *Clostridium difficile*-infections [35] is still in its infancy (Table 1). Most of these studies indicate a disease-specific body odour or a specific volatile organic compound (VOC)-pattern associated with metabolic changes secondary to an infection [36]. In case of an infection with a virus, VOCs are generated purely by the host cell, but for bacteria, VOCs are generated by the host and the bacteria respectively [36]. For many diseases the exact odour molecules that are recognised and indicated by dogs remain unknown. Disease-specific VOC-patterns have been identified in diseases such as asthma, several types of cancer, cystic fibrosis, diabetes mellitus, dental diseases, gastrointestinal diseases, heart allograft rejection, heart diseases, liver diseases, pre-eclampsia, renal disease, cholera and tuberculosis [36–38].

**Table 1:** Overview medical detection dog studies

Publication	Authors	Detection of	Study design	Sample material	Sample size	Results
Real-Time Detection of a Virus Using Detection Dogs [23]	Angle et al. (2016)	Bovine viral diarrhea virus	Randomised, blinded	Cell culture	n = 15	Sensitivity 91% Specificity 99%
Trained dogs identify people with malaria parasites by their odour [34]	Guest et al. (2019)	Malaria infection	Randomised, blinded	Body odour (socks)	n = 175	Sensitivity 72% Specificity 91%
Detection of Bacteriuria by Canine Olfaction [22]	Maurer et al. (2016)	Bacteriuria	Randomised, blinded	Urine	n = 687	Sensitivity near 100% Specificity above 90%
Using Dog Scent Detection as a Point-of-Care Tool to Identify Toxigenic Clostridium difficile in Stool [35]	Taylor et al. (2018)	Toxigenic *Clostridium difficile*	Randomised, blinded	Faeces	n = 300	Sensitivity 85% Specificity 85%
Olfactory detection of human bladder cancer by dogs: Proof of principle study [8]	Willis et al. (2004)	Bladder cancer	Randomised, blinded	Urine	n = 144	mean success rate 41%
Olfactory Detection of Prostate Cancer by Dogs Sniffing Urine: A Step Forward in Early Diagnosis [9]	Cornu et al. (2011)	Prostate cancer	Randomised, blinded	Urine	n = 66	Sensitivity 91% Specificity 91%
Key considerations for the experimental training and evaluation of cancer odour detection dogs: lessons learnt from a double-blind, controlled trial of prostate cancer detection [10]	Elliker et al. (2014)	Prostate cancer	Randomised, blinded	Urine	n = 181	Sensitivity 19% Specificity 73%
A Proof of concept: Are Detection Dogs a Useful Tool to Verify Potential Biomarkers Biomarkers for lung cancer? [11]	Fischer-Tenhagen et al. (2018)	Lung cancer	Randomised, blinded	Absorbed breath samples	n = 60	correct identification average 95%, correct negative indications average 60%
Accuracy of Canine Scent Detection of Non–Small Cell Lung Cancer in Blood Serum [12]	Junqueira et al. (2019)	Non–small cell lung cancer	Randomised, blinded	Blood serum	n = 10	Sensitivity 97%, Specificity 98%
Diagnostic accuracy of canine scent detection in early- and late-stage lung and breast cancers [14]	McCulloch et al. (2006)	Lung and breast cancer	Randomised, blinded	Breath	n = 169	*Lung cancer*: Sensitivity 99% Specificity 99% *Breast cancer*: Sensitivity 88% Specificity 98%
How dogs learn to detect colon cancer-Optimizing the use of training aids [15]	Schoon et al. (2020)	Colon cancer	Randomised, blinded	Faeces	n = 70	Average hit rate 84% Average false positive rate 12% (for new unknown samples)
Colorectal cancer screening with odour material by canine scent detection [17]	Sonoda et al. (2011)	Colorectal cancer	Randomised, blinded	Breath and faeces	n = 350	*Breath*: Sensitivity 91% Specificity 99% *Faeces*: Sensitivity 97% Specificity 99%
Cancer odor in the blood of ovarian cancer patients: a retrospective study of detection by dogs during treatment, 3 and 6 months afterward [16]	Horvath et al. (2013)	Ovarian cancer	Randomised, blinded	Blood plasma	n = 262	Sensitivity 97% Specificity 99%
Can Trained Dogs Detect a Hypoglycemic Scent in Patients With Type 1 Diabetes? [123]	Dehlinger et al. (2013)	Hypoglycaemia	Blinded	Skin odour	n = 24	Sensitivity 56% Specificity 53%
Dogs Can Be Successfully Trained to Alert to Hypoglycemia Samples from Patients with Type 1 Diabetes [42]	Hardin et al. (2015)	Hypoglycaemia	Randomised, blinded	Sweat	n = 56	Sensitivity 50%-88% Specificity 90%-98%
How effective are trained dogs at alerting their owners to changes in blood glycaemic levels?: Variations in performance of glycaemia alert dogs [18]	Rooney et al. (2019)	Hypoglycaemia	Not applicable	Breath and sweat	Not applicable	Median sensitivity 83%
Variability of Diabetes Alert Dog Accuracy in a Real-World Setting [19]	Gonder-Frederick et al. (2017)	Hypoglycaemia	Not applicable	Body odour	Not applicable	Sensitivity 57% Specificity 49%
Reliability of Trained Dogs to Alert to Hypoglycemia in Patients With Type 1 Diabetes [20]	Los et al. (2017)	Hypoglycaemia	Not applicable	Body odour	Not applicable	Sensitivity 36%
Dogs demonstrate the existence of an epileptic seizure odour in humans [21]	Catala et al. (2019)	Epileptic seizure	Pseudo-randomised, blinded	Breath and sweat	n = 5	Sensitivity 87% Specificity 98%
Canine detection of volatile organic compounds unique to human epileptic seizure [43]	Maa et al. (2021)	Epileptic seizure	Randomised, blinded	Sweat	n = 60	Probability of distinguishing ictal versus interictal sweat 93% Probability of canine detection of seizure scent preceded clinical seizure 82%

Canine medical scent detection appears more promising for infectious diseases than non-infectious diseases such as cancer, diabetes mellitus and epileptic seizures. Despite some initially promising medical dog scent detection studies, published data can vary significantly for the identification of cancer. Studies with trained sniffer dogs achieved very different results in the identification of different cancer types, such as bladder, prostate or ovarian cancer, lung and breast cancer as well as colorectal neoplasms. Diagnostic accuracies varied with sensitivities ranging from 19 to 99% and specificities from 73 to 99% when compared to histopathology [8–17]. Different sample materials were used for presentation, e.g. urine, blood, breath or faeces, which could explain the variability in findings. Another influencing factor that plays a role regarding the variability of the results is the lack of standardisation of training and the trainer bias, which may have a major influence on the training results of detection dogs [39]. More published data about cancer detection by dogs is reviewed elsewhere [40, 41].

Medical scent detection dogs have also been deployed for patients with diabetic mellitus. Identifying hypoglycaemic conditions is crucial for people with diabetes mellitus because of the potential severity of such a condition. A drop of blood is required to measure blood glucose, which is an invasive method that must be performed consciously and regularly. Not every patient is able to take blood themselves. The deployment of a hypoglycaemia sniffer dog is a non-invasive method, but satisfactory results have not been achieved. The researchers found sensitivities between 36% [20] and 88% [42] and specificities of 49% [19]–98% [42] compared to the standard method blood glucose measurement via blood glucose meter.

The prediction of an epileptic seizure could help the affected person to find a safe environment before the seizure begins or to take emergency medication. It is assumed that canines have the ability to detect an alteration of the body odour. Due to a high variability of the types and causes of epilepsy, it is still unknown which specific odour the dogs detect but chemical analyses could identify seizure-specific odour molecules [21]. In another study using sweat of persons with epilepsy, canines distinguished between interictal and ictal sweat with a probability of 93% and warned the individual before a clinical seizure occurred with a probability of 82% [43]. Some studies also report seizure alerting dogs that did not undergo any systematic training [44–46]. These dogs may detect specific odour-alterations as well as visual cues or behavioural changes of the person with epilepsy [44–46].

Studies including the detection of infectious diseases by dogs appear to be more promising. The training of detection dogs in the following studies was reward-based (based on positive reinforcement). Guest et al., 2019, performed a study for the detection of protozoal Malaria to develop a non-invasive screening method for infected individuals [34]. Even in asymptomatic children the dogs had a sensitivity and specificity of 72% and 91%, respectively. Previously worn nylon socks were presented to the two trained dogs. The results were higher than the threshold for WHO malaria diagnostics [34]. The training of dogs to identify bacterial infections like bacteriuria in urine [22] or *Clostridium difficile* in stool samples [35] also generated promising results. Maurer et al., 2016, trained dogs to improve strategies for detecting early stages of bacteriuria before the infection becomes serious. The dogs detected different pathogens (*Escherichia coli, Enterococcus, Klebsiella, Staphylococcus aureus*) with an overall sensitivity of close to 100% and specificity of above 90% [22]. For the detection of toxigenic *Clostridium difficile* in stool samples, two dogs were trained and achieved sensitivities of 78% and 93% as well as specificities of 85%, respectively. The aim of this study was to evaluate the dog method as a “point-of-care” diagnostic tool [35]. Lastly, it was also possible to train dogs to detect viral infections with bovine viruses [23] or with the coronavirus SARS-CoV-2 in various body fluids [6, 24–33] with high rates of diagnostic accuracy. Real-time methods for the identification of viral infections are often limited or not existing, especially for resource-limited environments. Angle et al., [23] examined the ability of two dogs to detect and discriminate bovine viral diarrhoea virus cell cultures from cell cultures infected with bovine herpes virus 1, bovine parainfluenza virus and controls with high rates of sensitivity and specificity (Table 1).

Recently, there is a rapid, growing body of evidence for detection dogs being used for identifying SARS-CoV-2 infected individuals [24–33]. In the SARS-CoV-2 detection dog studies, different sample material, study designs and dog breeds were used in different countries. Most of these studies achieved promising results (Table 2). The most common dog breeds used were Malinois, other shepherd breeds and Labrador Retrievers. These dogs are specifically bred for scent detection, selected for their scenting ability with an appropriate cognition and motivation behaviour making them popular breeds for biomedical detection [47]. The samples were collected initially mainly from hospitalised COVID-19 patients, but now as well as from asymptomatic and mildly symptomatic infected individuals with a variety of symptoms. Some researchers used distractors (samples from individuals suffering from other respiratory diseases than COVID-19) in the training and testing phases, which were slightly different from the target scent to better represent conditions in the field where other respiratory diseases different from COVID-19 will be to be also presented. A wide variety of human body fluids (saliva, tracheobronchial secretions, urine, sweat) as well as nasopharyngeal swabs, breath samples and masks or clothing were used as sample materials for presentation to dogs during training and testing [24–33]. Interestingly, dogs trained with saliva samples were also able to detect samples of infected individuals in sweat and urine without further training which is indicative for a successful generalisation process [25]. In detection dog training, it is important to pay equal attention to generalisation and discrimination. Generalisation means that after successful training, the dog also reacts to new, unknown stimuli that have similar odour properties to the training odour, whereas discrimination means the ability to distinguish between similar stimuli [48]. Without successful generalisation by the dogs, they would memorise the individual odours of the training samples individually and would have great difficulty recognising new samples as positive or negative. A lack of discrimination process would mean that the dogs would not only indicate the specific disease they were trained for, but would also indicate similar odours, e.g. respiratory diseases other than COVID-19, which would preclude the dog’s use as a screening method.

**Table 2:** Overview of SARS-CoV-2 detection dog studies

Country	Sample material	Number of sample presentations (test)	Results	
			Sensitivity	Specificity
France [26]	Sweat	n = 321	90% and 88%	90% and 85%
Germany [24, 25]	Inactivated saliva/tracheobronchial secretion	n = 1012	83%	96%
	Non-inactivated saliva	n = 2513	82%	96%
	Sweat	n = 531	91%	94%
	Urine	n = 594	95%	98%
Iran [29]	Nasopharyngeal	n = 80	65%	89%
	Masks and clothes	n = 120	86%	93%
Colombia [28]	Saliva/respiratory secretions	n = 9200	89%	97%
Brazil [6]	Not applicable	Not applicable	Not applicable	Not applicable
United Arab Emirates [30]	Axillary sweat	n = 1368	92%	96%
United Arab Emirates [32]	Sweat	n = 3290	83%	99%
Argentina [6]	Not applicable	Not applicable	93%	89%
Australia [6]	Not applicable	Not applicable	100%	95%
Lebanon [6]	Sweat	Not applicable	100%	92%
	Sweat (airport)	Not applicable	96%	90%
Chile [6]	Sweat	Not applicable	90%	97%
Finland [6]	Sweat, urine, saliva	Not applicable	100%	91%
Belgium [6]	Sweat	Not applicable	81%	98%
United Kingdom [31]	Breath and sweat	n = 2261	82%-94%	76%-92%
USA [27]	Saliva and urine	n = 59	11–22%	94–100%
	Urine		71%	99%
USA [33]	Breath	n = 160	Not applicable	Not applicable

All included studies were double-blinded and randomised

The various sample materials differ in the ease of collection, VOCs contained and infectivity, e.g. sweat or urine samples seem to be less infectious than saliva [49, 50]. Our own canine experience has shown that sensitivity and specificity of each dog appeared slightly different for each presented sample, but fairly similar overall for each bodyfluid [25]. The reason for this could be that not every dog learned the same VOC-pattern as being positive but slightly different ones. When working with detection dogs, it is not possible to know exactly to which specific VOCs the dogs were conditioned to. It is also unknown whether each dog had learned the same disease-specific VOC-patterns as being positive. Nevertheless, the study results of the different research groups indicate that all dogs could be successfully conditioned to a specific virus-induced odour, otherwise the results listed below could not have been achieved [24, 25]. Most studies have not pre-selected dogs, but this would be needed when using them as a diagnostic test, with only the best performing dogs being used.

Sensitivities in the different studies ranged from 65 to 100%, specificities from 76 to 99% (Table 2).

The training and test design in the various studies differed, but the dog training in all of them was based on positive reinforcement. As training and testing setup, most of the studies included the dogs working on a line-up with different numbers of samples presented. Sample material from SARS-CoV-2 positive individuals was used as target samples, negative controls were obtained from healthy individuals and only some groups also used distractors (sample material from individuals suffering from other respiratory diseases other than COVID-19) to train and test the detection dogs.

### Origin and composition of odours

What are dogs scenting when they identify an infected individual? The complex process of odour recognition starts with the development and composition of odours [51]. The majority of odours detected by dogs through inhaling are VOCs in different compositions residing in the air [51]. VOCs can differ in magnitude, volatility, and concentration. The odour concentration in the air correlates with the concentration of its source, volatility, the sources odour releasing surface area, the volume flow rate, ambient air movements and diffusion velocity within its source [52]. On top of that and depending on the materials in contact with specific odours, adsorption or absorption of VOCs occurs which is important for sampling and sample presentation for biomedical detection dogs. In general, liquids and plastic polymers absorb odours whereas surfaces like metal, glass, wood and cotton adsorb and release them [53].

The term ‘VOC’ describes atmospheric trace gases except for carbon dioxide and monoxide. Biogenic VOCs e.g. are isoprene and monoterpenes (most prominent compounds), as well as alkanes, alkenes, carbonyls, alcohols, esters, ethers and acids [52], have a strong odour and are produced as well as emitted by animals, plants and micro-organisms. The VOC-pattern of an organism is governed by VOC-producing cells or tissues and largely determined by its physiological or patho-physiological metabolism, the latter being subject to exogenous influences like infections, skin emanations or smokers’ breath [52]. Different diseases cause the emergence and emission of more or less specific VOC-patterns [36], which can be used as diagnostic olfactory biomarkers. Abd El Quader et al. [54] identified pathogen-related VOCs emanated from viral and bacterial cultures and Steppert et al., [55] found a difference in emanated VOCs between SARS-CoV-2 and Influenza-A infections in human breath. Various possibilities of measuring specific VOCs are existent, such as gas chromatography mass-spectrometric techniques (GC–MS) for identification and characterisation [36]. The diagnostic potential of scent detection dogs for VOC-based disease detection has been discussed recently [51].

VOCs are liberated from various tissues and body fluids. The most common body fluids or tissues for diagnostic testing are skin emanations, urine, blood, saliva and faeces differing in their VOC-composition [56]. Human bodies emit an extensive repertoire of VOCs that vary with age, diet, gender, genetics and physiological or pathological status and can be considered as individual attributes [36]. Pathological processes influence the body odour either by producing new VOCs or by changing the VOC-pattern which dogs may be able to detect [36]. During the training of biomedical detection dogs it is therefore important that dogs are not conditioned to the individual odours of the subjects or the environment were samples were produced (e.g. hospital smell) but learn the disease-specific odour (VOC-pattern) and successfully complete the generalisation process. It is also important to emphasize that the biochemical origins for some of the VOCs have not been completely elucidated until now.

### Neuroanatomy of olfaction

The anatomical construction of the olfactory system is highly structured in order to ensure efficient nasal odorant transport as well as respiratory airflow [57]. The sensory impression emerges through the olfactory system [58]. The substantial elements of the canine olfactory system are the outer nose with nares and nasal wings, nasal cavity, the olfactory epithelium with receptors, the vomeronasal organ, the olfactory bulb and the olfactory cortex of the cerebrum [58]. The bilateral nasal cavity is divided in the median plane by the nasal septum. Each side includes a nasal vestibule lined with cutaneous mucosa, a respiratory and an olfactory region, but also comprises a naso-, maxillo- (lined with respiratory epithelium and a small number of olfactory neurons) and ethmoturbinate (olfactory epithelium) to increase the olfactory mucosal surface, especially in macrosmatics [58, 59]. The three turbinates divide the nasal cavity’s chamber into three meatuses of the nose, whereby the ventral meatus is responsible for the respiration (inspiration and expiration). The dorsal meatus leads to the olfactory organ, whereas the middle nasal meatus terminates in the paranasal sinuses [58].

**Fig. 1:**
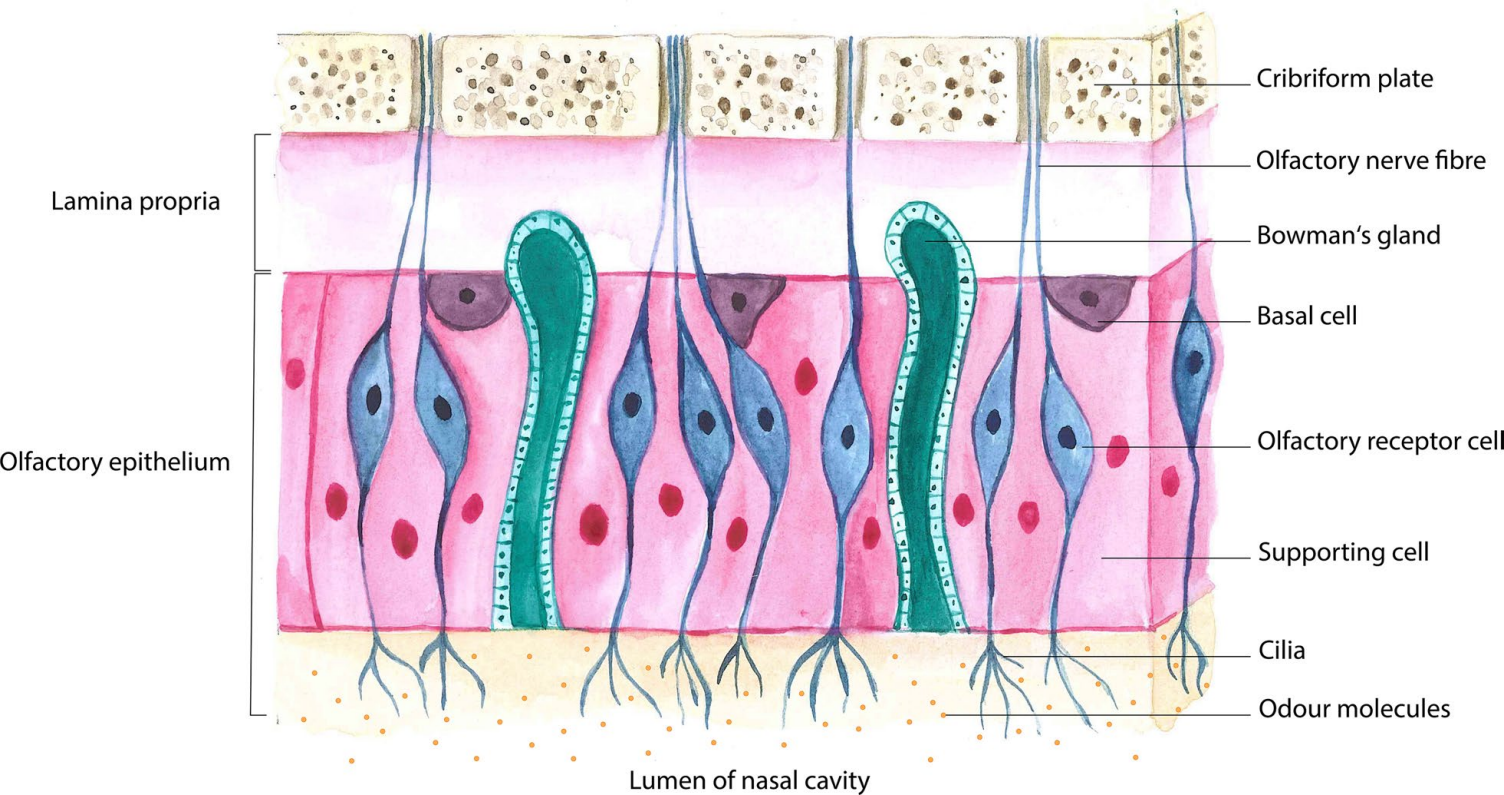
Schematic structure of the olfactory mucosa

Figure 1 shows the general structure of the olfactory mucosa. All components between the lumen of the nasal cavity and the cribriform plate are shown in simplified form. The nasal cavity lining has the function to separate odour molecules by their partition coefficients into the mucosa [60] and to create different flow dynamics in order to distribute odour molecules to the receptors, thus patterning the odorants [61]. Olfactory molecules in the nasal cavity lumen bind to olfactory receptors on the cilia of olfactory receptor cells embedded between supporting cells. The respiratory epithelium consists of a multi-row ciliated epithelium with goblet cells [58]. The olfactory epithelium implies a pseudostratified columnar neuro-epithelium [62] located next to the cribriform plate and lining the turbulate bones symmetrically in the nasal cavity [63] with millions of olfactory receptor cells (ORC) and olfactory receptors (OR), but also supporting sustentacular cells regulating the nasal mucous composition, isolating the ORCs and protecting the epithelium from inhaled potentially dangerous substances [64]. Moreover, basal cells are located adjacent to the lamina propria in the olfactory epithelium and comprise Bowman’s glands in the lamina propria whose secretion builds a mucous layer in combination with the sustentacular cells’ substances which maintain nasal humidity and capture odorants [58, 62]. The lamina propria itself is adjacent to the bony lamina cribrosa, which is traversed by olfactory nerve fibres. The regular olfactory perception depends on this area [64].

**Fig. 2:**
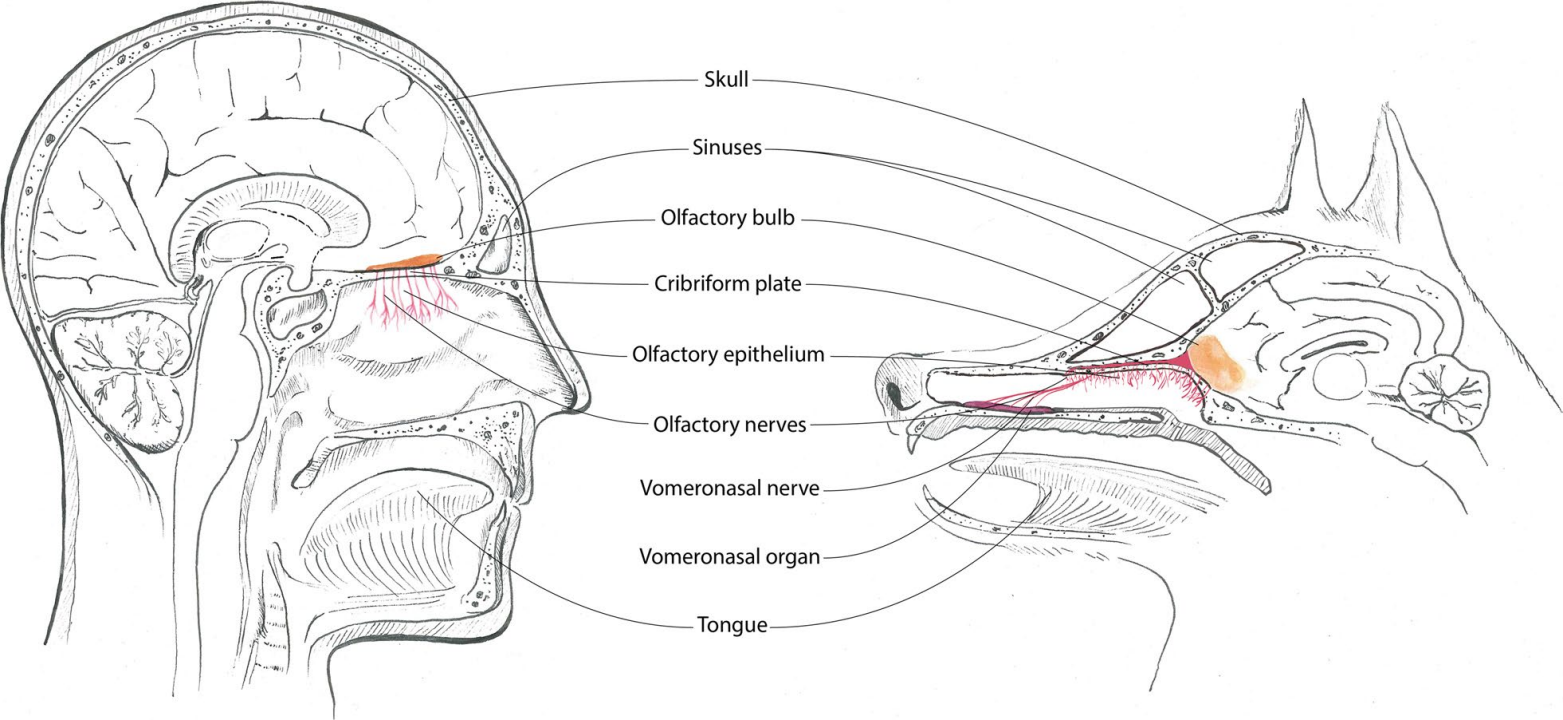
Schematic structure of the olfactory system in dogs and humans

An additional olfactory system can be found in the vomeronasal organ of dogs (Fig. 2).

Via unique airflow patterns, environmental odorants selectively bind to the ORs to initiate odour perception [59]. The ORC is a bipolar neuron: the dendrite extends in the direction of the olfactory epithelium (nasal cavity) and terminates with the ORs located in the membrane of multiple cilia in the mucous layer, whereas the axons of all ORCs build the olfactory ‘nerve’ (fila olfactoria) passing through the cribriform plate and to the olfactory bulb. The complex structure gives dogs the ability to detect an enormous quantity of different odour molecules with subtle shape, size or stereoisomeric differences [65, 66]. The exact sequence of the olfactory process at the molecular level has been reviewed elsewhere [58]. The glomeruli approach dendrites of mitral cells and tufted cells whose axons constitute the lateral olfactory tract that conducts the signal to the piriform cortex and project it to the olfactory cortex in the medial temporal lobes [64].

The olfactory cortex receives sensory signals from the olfactory bulb. The processing of olfactory signals in the brain is also beyond the scope of this review and can be found elsewhere [58]. An overview of important olfactory characteristics of dogs is presented in Table 3.

**Table 3:** Olfactory characteristics of dogs

Characteristics	Dog
Airflow	A sniff creates unique unidirectional laminar airflow patterns to transport environmental odorants to the olfactory epithelium [122]
Size of olfactory mucosa	95–126 cm^2^ (German Shepherd) [124]
Olfactory genes in genome	> 1000; 80% functional receptor genes and 20% pseudogenes [125]
Amount of olfactory receptor cells (ORCs)	200–300 million in nasal cavity [58, 59]
Cilia per ORC	20 to 100 cilia per cell [58]
Extent, shape and position of olfactory bulb	Proportionally larger than in humans and prominently at the ventro-rostral area of the brain [126]

**Fig. 3:**
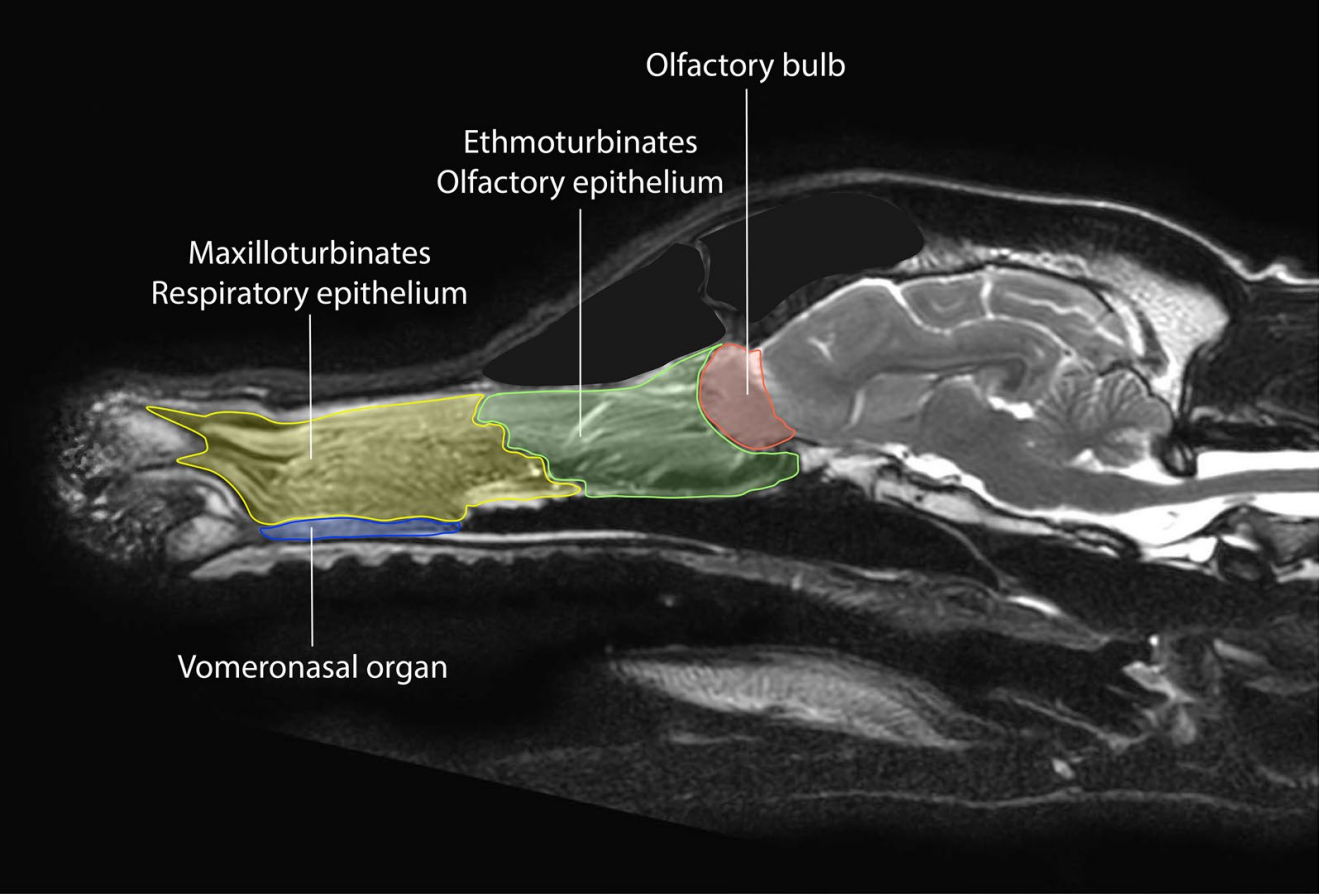
Sagittal magnetic resonance imaging highlighting the inner structures of the olfactory system. The blue area represents the vomeronasal organ, the respiratory epithelium in the maxilloturbinates is highlighted in yellow, the olfactory epithelium in the ethmoturbinates near the lamina cribrosa is shown in green, and the red area contains the bulbus olfactorius

Figure 2 shows a comparison of the olfactory system between dog and human. Components of the olfactory system are shown in colour. Particularly noticeable are the differences in extent, shape and position of the olfactory bulbs and the vomeronasal organ, which is present only in dogs but not in humans. It is located bilaterally symmetric on the ventro-rostral bottom of the nasal cavity behind the canine teeth and associated to the nasal and oral cavity. Its sensory epithelium detects mainly pheromones and non-volatile molecules for intra-species-specific communication and reproduction. The transmission follows a separate pathway directly to the hypothalamus [59, 67]. Figure 3 presents important inner structure of the olfactory system.

### Physiology of olfaction and fluid dynamics

**Fig. 4:**
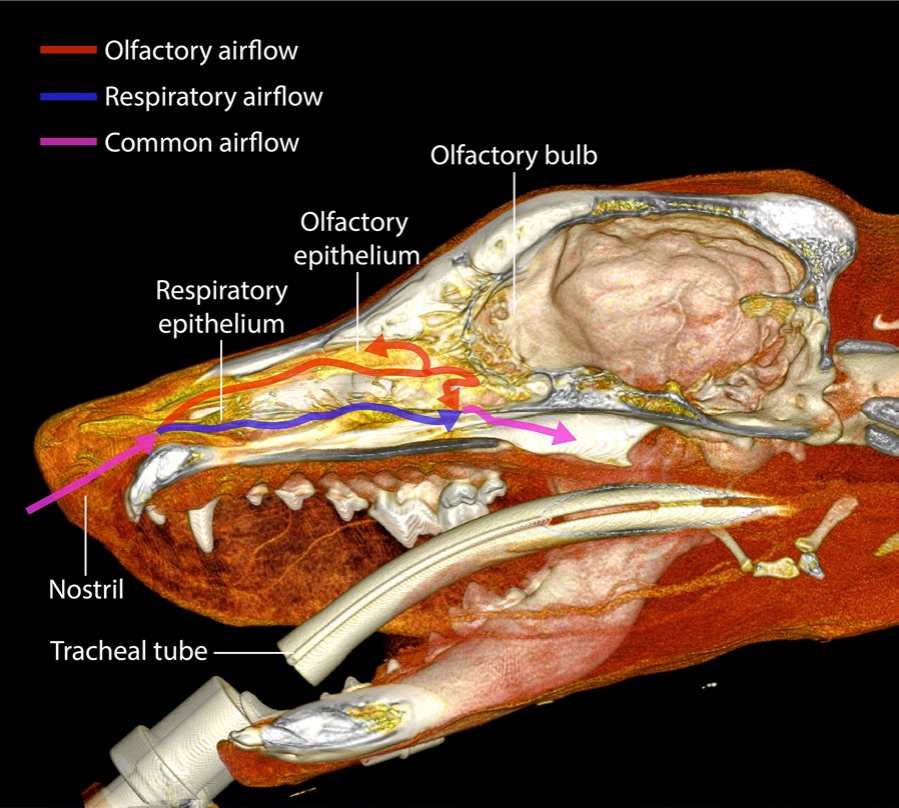
Three-dimensional computed tomographic reconstruction of a canine skull. The arrows represent the airways, with the pink arrow showing the common airflow and the red and blue arrows showing the olfactory and respiratory airflow, respectively. The nostrils, the olfactory and respiratory epithelium as well as the olfactory bulb, and the tracheal tube are labeled. During inhalation the air flows from the nares and the nasal vestibule to the maxilloturbinates, then into the ethmoturbinates and the paranasal sinuses towards the pharynx [122]. There is a major difference between breathing and sniffing in dogs. While breathing, most of the inspired air flows through the nasopharynx into the lungs but only a small percentage (12–13%) reaches the olfactory areas [59]. The sniffing process generates external (outside the nostrils) and internal (within the nasal cavity) fluid dynamics. The ambient air is inhaled from the front and exhaled to the side for efficient odorant sampling, whereas each nostril samples separately. A sniff is the first critical step of the olfactory process with the function of generating unique unidirectional laminar airflow patterns to transport environmental odorants into the nasal cavity to the olfactory epithelium [122]. Furthermore, sniffing increases odour sensitivity and affects the intensity of odorants [60, 90]

Animals use olfaction to find and select nourishment or prey [68, 69], for recognition of social partners, predators or environmental toxins as well as for orientation and communication [36, 70]. Body odours function as indicators of the metabolic status of individuals [36]. The canine’s scent detection ability limit for VOCs has a reported range of parts per million and parts per trillion [71]. Different types of airways in the canine nasal cavity are shown in Fig. 4.

In the cognition process of various stimuli, hemispheric specialization takes place [72]. For the olfactory pathway, it means, that olfactory stimuli ascend ipsilaterally from the detection place in the nasal cavity to the place of perception in the olfactory cortex [72]. Dogs use preferentially the right nostril to detect conspecific arousal or novel odours, transmitting sensory input to the right cerebral hemisphere to process alarming stimuli. The left nostril is preferentially used to sniff non-aversive, familiar and heterospecific arousal odours as well as target odours by detection dogs [73].

Current literature is not clear about breed-specific olfactory capabilities and discusses the influence of genetic polymorphism in comparison to behaviour and trainability [47, 74, 75].

### Factors impacting olfaction

There are several circumstances which can affect the olfactory sense of dogs [58]. Some are of physiological origin, others are pathological. Especially when working with biomedical detection dogs, it is important to know these factors and adjust the working conditions for the dogs as best as possible.

Physiological variation in the olfactory capability is most frequently caused by differences in *genetics*. In general, macrosmatic animals have an olfactory gene array of greater extent, much larger than microsmatics. A comprehensive overview of the genetic influence on the sense of smell in dogs can be found in the according literature [76–81].

Differences in the olfactory capabilities of different dog breeds and wolves are also described. Polgàr et al., [47], compared detection abilities of dog breeds selected for scenting abilities, dog breeds for other purposes, brachycephalic dog breeds and hand-raised wolves. As a result, the breeds selected for odour work (e.g. Shepherd Dogs or Labradors) and in some tests even the wolves performed better than the other dogs and the short-nosed breeds.

Secondly, the environmental conditions influence the odour sensing abilities. Relative humidity and barometric pressure may directly affect olfaction, besides their effects on odour emergence and movement itself, while heat has only indirect effects [58, 82–84]. Acclimatization to the environment, physical fitness and an adequate hydration state can prevent heat stress of the dogs [84].

Age can influence the sensory process [85]. Olfaction and its cognition are impacted by age in humans [86] as well as in dogs [58]. Age affects various functional parts of the olfactory system in dogs at an age of older than 14 years [86, 87].

Conditioning, training, and management play a major role for the use of dogs as detection dogs [58]. Exercise and condition deficiencies are described as physical stressors which may affect the olfaction in canines directly or indirectly [58]. Physical exercise affects the olfaction of detection dogs by decreasing finding rates, especially in dogs with poor physical conditions [88, 89]. As a result, a working dog should be well trained to have an optimal physical condition [90]. Scent detection training techniques can improve odour sensitivity and discrimination [89–92]. Housing and general management may influence the dogs’ detection work as well by affecting the learning capability. Lower stress levels due to social contact and an enriched, secure environment were shown to enhance cognitive performance [87]. Positive rewards with particularly tasty treats as well as a specific toy in some dogs increase working motivation of dogs whereas aversive training methods decrease motivation and have also negative effects on their physical and mental health [93].

Hydration [84], nutrition [88, 89], and the microbiome [58] of dogs manipulate the olfactory sense as well. As mentioned above, heat stress influences olfaction due to provocation of panting but dogs are able to develop heat tolerance by establishing an adequate hydration status [84, 89].

The nutritional factor influencing the olfactory sense of dogs includes the feeding time, amount of food per meal, ingredients like the fat/protein ratio and the fat source [88, 89, 94–98].

Various diseases and medication can affect the olfaction of dogs and lead to hyposmia or anosmia. More information on hyposmia can be found elsewhere [58, 99].

Diseases or disorders potentially leading to hyposmia or anosmia in humans and potentially in dogs [100] are congenital and neurodegenerative diseases [86, 101], metabolic, endocrine (hyperadrenocorticism, diabetes mellitus, and hypothyreoidism [101]) and neurological diseases like nasal/brain tumours, granulomatous meningoencephalitis or head trauma [102], general inflammation and systemic diseases, exposure to dust and toxic chemicals/materials, uraemia and blood flow changes as well as the hydration state [103]. Different infections of the upper respiratory tract, e.g. SARS-CoV-2-infections, can also cause anosmia in humans [104]. Whether dogs play a role in the infection incidence of SARS-CoV-2 is controversially discussed in the literature and there is no evidence that dogs might be affected by anosmia through a SARS-CoV-2-infection so far, but it also cannot be completely excluded by now [105–107]. Dog-specific viral diseases like canine distemper virus and canine parainfluenza virus [108] cause conductive hyposmia by generating nasal inflammation and increasing mucous secretion and result in vascular congestion that alters the air flow. Furthermore, allergic rhinitis and turbinate engorgement caused by hypocapnia, cold air, irritating chemicals or an increased parasympathetic tone result in olfactory decrease or loss [109].

Some pharmaceuticals used in human medicine are also applicable for dogs and may potentially have similar effects in dogs [110]. Only specific effects of steroids, antibiotics and anaesthetics on the dog’s olfaction are documented in the scientific literature at this time [58]. Other medications potentially endangering dog's olfaction are described elsewhere [58, 111–116].

## Discussion

The special structure and physiology of the canine olfactory system contain a huge potential of olfactory power [58]. The dog’s sense of smell is mainly used to attract prey and to perceive the environment but could also be promoted and meaningfully used by humans for biomedical purposes. Since the vomeronasal organ (VNO) has an important function in intra-species communication or the detection of pheromones and is capable of processing a wide variety of molecules, it may be possible that direct detection of viruses or viral proteins (not VOCs) by the VNO occurs, thus representing a different mechanism of odour perception. However, this is only a hypothesis and has not yet been proven.

Various diagnostic studies have addressed the detection of different diseases by dogs. Despite the promising results of the scent detection dogs, this method is only marginally or not used in the field of human medicine. The majority of medical professionals continues to rely on diagnostic standard methods although the canine medical detection method achieved equal or even higher rates of diagnostic accuracy. For example, electronic noses have a limit of detection of 100 to 400 parts per billion (ppb) (1 × 10^–7^) [117] whereas the olfactory detection threshold of dogs is lower than 0.001 ppb (1 × 10^–12^) [71], so they surpass this technology by far. But inconsistent findings and the complexity of this research area prevents the practitioners from including this method in their daily routine. Moreover, a medical device or health technology requires an approval by national health organizations before permission for usage is granted. For such approval, ethical, social, organisational, and legal aspects are assessed alongside technological, economic and safety aspects, as well as clinical effectiveness [5].

Other limitations of the medical scenting dog method is the current lack of standardisation of the training and deployment of biomedical detection dogs (although this has been tried in several detection dog studies [5, 118]) as each dog has an individual character, an individual training level or training requirements, and there are several different breeds with a variation of characters and olfactory thresholds. But there are legislated guidelines and commission regulations for the use of explosive detection dogs, which could and should be used in a modified form for biomedical detection dogs as well [119, 120]. In addition, dogs are living creatures with varying detection performances at different times. Moreover, the training condition has to be maintained with regular training, unlike in machines. Variation of detection accuracy may be caused by failure in odour conditioning, lack of motivation, inappropriate training methods (e.g. alternative forced choice without blank trials [5]) or other confounding factors [40]. A test run at the beginning of the detection work could reduce the error frequency of the dogs. The disease detection dog studies differ in terms of experimental setup, sample material (urine, breath, blood, saliva, faeces, sweat/body odour) and sampling method, individual dog characteristics, dog training methods and evaluation strategies of the results. For some diseases like different cancer types, the canine method seems to be not very useful due to the need of reliable identification of early, preclinical stages that require surgical intervention. To reliably diagnose a certain disease, laboratory testing or equivalent methods still have to be performed because of the fact that the canine method is not generally accepted and approved.

The advantages of the canine method are especially the non-invasiveness, speed, nearly immediate results, effectiveness of testing, cost effectiveness, mobility, high sensitivity and specificity of the dogs’ noses, safety for persons to be tested and persons performing the test (dog handlers), the simplicity and security of the sampling, testing procedure, specimen storage and evaluation of the results. Acquisition and training of a medical detection dog is maybe less cost-intensive than the purchase of expensive high-tech equipment. The sample collection requires no special abilities of the performing person and is not associated with any health risk for the patient due to the non-invasiveness in contrast to some standard methods. The samples can be preserved for some time and presented to several dogs which may increase the diagnostic accuracy. The sample material can be adapted to the disease to be tested (disease-specific VOCs) and even varied if necessary to reduce or eliminate the infection risk of the operating persons and dogs. The training period for emerging diseases is much less time-consuming than inventing a new technological test method. If the training period is once completed, the testing procedure is easy and time saving. While testing, there are four possibilities for the dogs to respond to the presented samples: True positive means, the dog correctly indicates a disease-positive sample; false positive means, the dog incorrectly indicates a negative control or distractor sample; true negative: the dog correctly does not indicate a negative control or distractor sample; and false negative: the dog incorrectly does not indicate a positive sample. For evaluation of the results and assessment of the diagnostic accuracy, the use of contingency tables can be useful. For testing of unknown samples, the possible indications are of binary character (disease-positive or -negative). The effectiveness of the dog method is also a great advantage. Dogs can screen large amounts of people in a short time with high rates of diagnostic accuracy. After a successful training phase, the dogs can be deployed in any setting or terrain, whereas most technological methods require standardised environmental conditions to function reliably, highlighting mobility as another meaningful advantage of the dogs. In summary, this method has promising potential for the effective detection of various infectious and non-infectious diseases after major limitations have been eliminated. Especially in countries with a lack of access to high technology screening methods or as a preliminary mass screening for infectious diseases at major events or airports the canine method has a huge potential.

## Conclusion

The use of biomedical detection dogs has many advantages and potential, but also some limitations. The literature shows that detection dogs can be considered as a screening method, especially for infectious diseases but may not be considered as a substitute for standard diagnostic methods until standardised and validated. In order to use biomedical detection dogs as an approved screening method for disease detection, the following issues need to be addressed: Standardisation of training and deployment techniques (ensuring generalisation to specific disease stages, symptomatic and asymptomatic patients), reproducibility within and between detection dogs, and (re-)certification by an official body. At this time, it should be recognised as an additional non-invasive, rapid diagnostic tool to effectively detect early stages of specific diseases in great confluences of people. Additional research is necessary to create a standardised, operationally viable system for canine olfactory detection of various human diseases. In addition, the ability of dogs to be able to discriminate between healthy and diseased patients can support identification of diseases in which VOCs could be characterised, e.g. via GC–MS like in Sethi et al. [121] for the development of different VOC based test systems.

## Data Availability

Data sharing is not applicable to this article as no datasets were generated or analysed during the current study.

## Abbreviations

VOC: Volatile organic compound
COVID-19: Coronavirus disease 2019
OR: Olfactory receptor
ORC: Olfactory receptor cell
ppb: Parts per billion
